# Generation of Polyclonal Antibodies Against Sabin Poliovirus D- and H-Antigens and Their Application in ELISA

**DOI:** 10.3390/vaccines13101022

**Published:** 2025-09-30

**Authors:** Anna Zyrina, Anna Shishova, Irina Tcelykh, Igor Levin, Olga Shmeleva, Nadezhda Borisenko, Maya Ermakova, Sergey Ivanov, Anastasia Kovpak, Vladislav Vasilenko, Yuliya Rogova, Alla Zhitkevich, Nikita Khabibullin, Yury Ivin, Anastasia Piniaeva, Alexandra Siniugina, Aydar Ishmukhametov

**Affiliations:** 1 Chumakov Federal Scientific Center for Research and Development of Immune-and-Biological Products of Russian Academy of Sciences (Institute of Poliomyelitis), Moscow 108819, Russia; 2 Institute for Translational Medicine and Biotechnology, First Moscow State Medical University (Sechenov University), Moscow 117418, Russia

**Keywords:** Sabin poliovirus strains, rabbit polyclonal antibodies, ELISA test, D- and H-antigens, polio vaccines

## Abstract

Background/Objectives: The World Health Organization (WHO) recommends the use of attenuated Sabin strains for the production of inactivated poliovirus vaccine (IPV), offering improved biosafety while retaining immunogenicity. To better characterize the antigenic composition of Sabin strain-based IPV (sIPV), including both the protective D-antigen and the non-protective H-antigen forms, we developed a method for purifying D- and H-antigens forms. Methods: D- and H-antigens of poliovirus Sabin strains types 1, 2, and 3 were purified using gradient ultracentrifugation and used to generate antigen-specific polyclonal antibodies. Results: The generated polyclonal antibodies demonstrated high specificity with neutralizing titers of antibodies against Sabin type 1 poliovirus—1:2048, against Sabin type 2 poliovirus—more than 1:2048, against Sabin type 3 poloivirus—1:2048. Conclusions: This antigen-specific antibody approach provides a valuable tool for routine quality control in sIPV manufacturing, enabling accurate quantification of immunogenic components and detection of potentially immunogenic degradation products during vaccine storage and distribution. Antibodies to the D-antigen allow assessment of immunogenic, neutralizing epitopes, while antibodies to the H-antigen provide a tool for detecting non-neutralizing components. This antigen-specific antibody approach offers a valuable tool for studying the antigenic structure of sIPV and for improving the accuracy of ELISA-based antigen quantification.

## 1. Introduction

Poliomyelitis is an acute infectious disease caused by one of the three poliovirus serotypes (types 1, 2, or 3). Prior to the introduction of vaccination, nearly all children contracted poliovirus, and approximately 1 in 200 infections resulted in irreversible paralysis. However, the implementation of widespread immunization programs has led to significant progress in controlling the disease [1]. Nevertheless, the emergence of virulent circulating vaccine-derived polioviruses (cVDPVs) from oral Sabin vaccines remains a concern, as they can cause vaccine-associated paralytic poliomyelitis in a small proportion of recipients [2,3]. This highlights the need for improved methods to assess vaccine quality [4].

Immunologically, poliovirus particles of types 2 and 3 are classified into two native forms: D-antigen, representing infectious, mature virions, and H-antigen (is also called C-antigen), representing non-infectious, immature particles [5]. D-antigen particles are composed of 60 copies of four capsid proteins: VP1, VP2, VP3, and VP4 (with VP4 located on the inner surface of the capsid) [6]. VP2 and VP4 result from the cleavage of the precursor protein VP0 [6,7,8]. In contrast, H-antigen particles lack genomic RNA and consist of VP0, VP1, and VP3 [9]. Only D-antigen particles are thought to be capable of inducing neutralizing antibodies, making their quantification essential for evaluating the immunogenic potency of poliovirus vaccines [10,11].

However, while D-antigen particles are critical for generating neutralizing antibodies, H-antigen particles can still play a role in modulating immune responses [5,12,13].

Also D-antigen can be converted to “imitative” form of H-antigen by various environmental stress conditions, including high temperatures [14,15]. For example in the article Rezapkin G, etc., D-antigen was heat for 1 h at 56 °C to form H-antigen [16].

Among the available methods for quantifying D-antigen units in poliovirus serotypes 1, 2, and 3, the sandwich enzyme-linked immunosorbent assay (ELISA) is the most widely employed due to its sensitivity and adaptability [17,18]. In such assays, the specificity of antibodies is critical, as cross-reactivity with H-antigen can compromise the accuracy of results [16,19]. 

It is noteworthy that many laboratories rely on monoclonal antibodies in D-antigen ELISA [20,21]. However, targeting a single epitope may not adequately reflect the overall antigenic activity of the vaccine. Although numerous neutralizing epitopes have been identified using monoclonal antibodies, there is still limited knowledge about specific epitopes that confer protective immunity. While combining multiple monoclonal antibodies may offer broader epitope coverage, it also increases assay complexity and cost [16,22].

To address these limitations, we utilized polyclonal antibodies raised against attenuated Sabin strains types 1, 2, and 3, enabling recognition of a broad spectrum of viral epitopes. Previous studies have demonstrated that inactivated poliovirus particles can undergo epitope modification or partial destruction, which may reduce the binding efficiency of monoclonal antibodies—further supporting the use of polyclonal antibodies [23]. This reaffirms the advantage of using polyclonal antibodies.

The selection of attenuated Sabin strains was motivated by the WHO’s recommendation to transition IPV production from wild-type to Sabin strains for enhanced biosafety [24,25]. Additionally, antigenic differences between wild and attenuated strains may influence antibody binding and vaccine performance [26].

In this study, we adapted our poliovirus purification method for isolating purified D- and H-antigens of Sabin strain polioviruses types 1, 2, and 3. These antigens were subsequently used to immunize rabbits, resulting in the production of specific polyclonal antibodies. The resulting antibodies are suitable for ELISA-based quantification of both D- and H-antigens in vaccine products and stability testing.

## 2. Materials and Methods

### 2.1. Purification of D- and H-Antigens of Poliovirus Strain Sabin Types 1, 2 and 3

FSUE “Institute of Poliomyelitis and Viral Encephalitis named after M.P. Chumakov” in October 1999 received 15 ampoules of the WHO Vero RCB 10–87 of the Vero cell line. Directly from the cell suspension of vial No.0519 of this bank (without making the seed bank of the Manufacturer’s cell line), a working bank of the Vero cell line (Vero WCB) was prepared at passage 139. In this study, the Vero WHO RCB 10–87 cell line (passages 141–149) was used for poliovirus production. Poliovirus strains Sabin type 1 (LSc 2ab), type 2 (P712 Ch 2ab), and type 3 (Leon 12a1b) were used for the antigen production. Vero cells (inoculation densities 0.2 ± 0.05 × 10^6^ cells/mL) were cultivated in single-use bioreactors (Cytiva, Marlborough, MA, USA and Sartorius Stedim, Aubagne, France) in EMEM (FSASI “Chumakov FSC R&D IBP RAS” (Institute of Poliomyelitis, Moscow, Russia) with fetal bovine serum (FBS, 5%, Gibco, Thermo Fisher Scientific, Waltham, MA, USA, cat. #10091148) on microcarriers Cytodex 1 (Cytiva, Marlborough, MA, USA) with concentration 3 g/L). To ensure optimal culture conditions, dissolved oxygen (DO), temperature, and pH were maintained at 70%, 37 °C, and 7.2, respectively. After reaching confluency, the Vero cell monolayer was washed with Hank’s buffer solution. Then, buffer solution was changed to M199 (FSASI “Chumakov FSC R&D IBP RAS”, Moscow, Russia). The cells were infected with 0.01–0.1 TCID_50_/cell MOI. Vero cells were incubated with the virus at 34 °C until complete monolayer degeneration was observed.

Virus particles produced in Vero cells were used as antigens for immunization. To obtain crude virus preparations, culture supernatants were cleared of cell debris by centrifugation at 17.050 rcf for 30 min at 4 °C using an Optima XPN-100 ultracentrifuge (Beckman Coulter, Brea, CA, USA). The clarified supernatant was then subjected to ultracentrifugation through a 3 mL 30% sucrose cushion (30% sucrose [cat. #S0389, Sigma-Aldrich, St. Louis, MO, USA], 1 M NaCl [cat. #S9888, Sigma-Aldrich, St. Louis, MO], and 20 mM Trizma, pH 7.5 [cat. #T7818, Sigma-Aldrich, St. Louis, MO]) at 106.559 rcf for 4 h at 4 °C. The resulting pellet was resuspended in 3 mL of 20 mM PBS (pH 7.2, FSASI “Chumakov FSC R&D IBP RAS” (Institute of Poliomyelitis)).

To remove residual lipids, 1.5 volumes of trichlorotrifluoroethane (CFC-113, cat. #48411, Sigma-Aldrich, St. Louis, MO) were added to the virus suspension, followed by vigorous mixing for 2–3 min on a vortex. The mixture was centrifuged at 986 rcf for 5 min at 4 °C, and the upper aqueous phase containing the virus was collected (Eppendorf Research, London, UK, 5810R).

Further purification was achieved by ultracentrifugation in a cesium chloride density gradient. Specifically, 3.265 mL of virus suspension was mixed with 1.735 mL of saturated cesium chloride solution (cat. #15554, Serva, Heidelberg, Germany; density 1.34 g/cc) and centrifuged at 181.799 rcf for 16–18 h at 4 °C (Optima XPN-100, Beckman Coulter). This procedure resulted in the formation of two distinct visible bands: the lower band corresponding to D-antigen and the upper band to H-antigen. Fractions of 200–300 μL were collected in wells of a 96-well plate after piercing the bottom of the tube with a needle from a disposable syringe. To identify protein-rich fractions, 3 µL of each collected fraction was applied to a nitrocellulose membrane and stained with Ponceau S solution. Ponceau S solution was poured onto the membrane for 30–40 s and the membrane was washed with water. The fractions with the highest protein content were pooled.

Desalting of antigen-containing fractions was performed by gel filtration chromatography on Bio-Gel P-2 columns (cat. #1504118, Bio-Rad, Hercules, CA, USA) using 20 mM PBS (pH 7.0) as the mobile phase.

Generalized scheme for purification of D- and H- antigens (Supplemental Figure S1).

As a result, five antigen preparations were obtained: D-antigen from Sabin type 1; D- and H-antigens from Sabin types 2 and 3. These were designated as Sabin 1, Sabin 2D, Sabin 2H, Sabin 3D, and Sabin 3H, respectively.

### 2.2. Transmission Electron Microscopy

Antigen-containing fractions were adsorbed onto glow discharged Carbon/Formvar-coated copper grids (Formvar/Carbon 200 Mesh, 3–4 nm carbon, FCF200-CU-SB, Electron Microscopy Sciences, Hatfield, PA, USA) for 5 min. The grids were then washed with 1 M EDTA (FSASI “Chumakov FSC R&D IBP RAS” (Institute of Poliomyelitis)), followed by staining with 2% uranyl acetate for 2 min (FSASI “Chumakov FSC R&D IBP RAS” (Institute of Poliomyelitis)), air-dried and examined in a transmission electron microscope JEM-100C (Jeol, Tokyo, Japan), magnification: X30000. The size of the obtained viral particles was determined using BioVision 4.0 (West Medica, Wiener Neudorf, Austria).

### 2.3. SDS-PAGE Under Denaturing Conditions

Poliovirus antigen preparations were analyzed by sodium dodecyl sulfate polyacrylamide gel electrophoresis (SDS-PAGE) in 20% polyacrylamide gels under denaturing conditions, in accordance with OFS.1.2.1.0023.15 Polyacrylamide Gel Electrophoresis, State Pharmacopoeia of the Russian Federation XIV, Volume 1. Electrophoresis was performed using a Mini-PROTEAN Tetra Cell system (Bio-Rad, USA). Protein bands were visualized by staining with Coomassie Brilliant Blue.

### 2.4. Lowry Assay

Protein concentrations in antigen and antibody samples were determined using the Lowry assay, in accordance with OFS.1.2.3.0012.15 Determination of Protein, State Pharmacopoeia of the Russian Federation XIV, Volume 1. Optical density at 750 nm (OD750) was measured using a Multiskan FC microplate spectrophotometer (ThermoFisher, Waltham, MA, USA). Bovine serum albumin (BSA; FSBI “SCEEMP” of the Ministry of Health of Russia) was used as a standard and serially diluted to final concentrations ranging from 200 to 10 µg/mL. A standard calibration curve was constructed in Microsoft Excel by plotting the average blank-corrected absorbance values at 750 nm against BSA concentrations (µg/mL), and was subsequently used to calculate the protein concentrations of the test samples.

### 2.5. Preparation of Rabbit Polyclonal Serum Against D- and H-Antigens of Poliovirus Strain Sabin Types 1, 2 and 3 and IgG Purification

Five rabbits of the Soviet Chinchilla breed were immunized twice with the obtained antigens Sabin 1, Sabin 2D, Sabin 2H, Sabin 3D, Sabin 3H, respectively (concentration range 170–560 µg/mL). During primary and second immunization, pure antigen was injected subcutaneously in a volume of 1 mL mixed with 300 μL complete Freund’s adjuvant (Calbiochem Research Biochemicals, Merck KGaA, Darmstadt, Hesse, Germany). A 21-day interval was maintained between immunizations. Following each immunization, control serum samples were collected no earlier than 14 days post-immunization, after which a neutralization assay was conducted. Upon reaching satisfactory titers of neutralizing antibodies (not less than 1:1024 for the D-antigen), a total blood sampling was carried out. IgG (against antigens Sabin 1, Sabin 2D, Sabin 2H, Sabin 3D, Sabin 3H) were isolated and purified from each serum sample by affinity chromatography on HiTrap columns filled with Protein G HP (cat #17-0405-01, Cytiva). Antibodies were eluted with 0.1 M Glycine-HCl buffer pH 2.7 (FSASI “Chumakov FSC R&D IBP RAS” (Institute of Poliomyelitis)) and pH immediately was adjusted to pH 7 by adding 1 M Tris-HCl pH 9 (FSASI “Chumakov FSC R&D IBP RAS” (Institute of Poliomyelitis)).

### 2.6. Neutralization Assay

The identification of titers of neutralizing antibodies was performed using a neutralization assay according to the standard WHO protocol [27].

### 2.7. Biotin-Conjugate Preparation

For storage purposes, purified antibodies were dialyzed into 0.1 M carbonate–bicarbonate buffer (cat. #C3041, Sigma-Aldrich, St. Louis, MO). For ELISA applications, a portion of these antibodies was conjugated with (+)-biotin N-hydroxysuccinimide ester (cat. #35013-72-0, Sigma-Aldrich, St. Louis, MO). All antibody preparations were adjusted to a concentration of 2 mg/mL. To achieve a biotin-to-IgG molar ratio of approximately 5:1, 300 µL of 1 mg/mL biotin ester (dissolved in DMSO) was added to each 10 mL of purified antibody solution. The conjugation was carried out according to the manufacturer’s instructions. Following biotinylation, the conjugated antibodies were dialyzed into 20 mM phosphate-buffered saline (PBS), pH 7.0.

### 2.8. ELISA Procedure

High-binding 96-well plates (Corning, NY, USA, cat. #9018) were coated with 90 µL of type-specific polyclonal rabbit IgG (concentration in range of 3–5 mg/mL, diluted 1:500 in 0.05 M carbonate–bicarbonate buffer, cat. #C3041, Sigma-Aldrich, St. Louis, MO) and incubated overnight at 4 °C. The following day, wells were blocked with 200 µL of blocking buffer (20 mM PBS containing 1% FBS, Gibco, cat. #10091148) for 1 h at 37 °C to prevent nonspecific binding. After each step, plates were washed twice with washing buffer (20 mM PBS with 0.05% Tween-80, cat. #P1379, Sigma-Aldrich, St. Louis, MO).

Next, 90 µL of D- or H-antigen diluted in dilution buffer (20 mM PBS, 0.05% Tween-80, and 1% FBS) was added to each well in a three-step dilution series. Wells containing dilution buffer alone served as background controls. Plates were incubated for 2 h at 37 °C.

Following this, 90 µL of biotin-conjugated polyclonal rabbit IgG (concentration in range of 1.2–1.9 mg/mL diluted 1:200 in dilution buffer) was added and incubated for 1 h at 37 °C. Then, 90 µL of streptavidin–peroxidase conjugate (cat. #S5512, Sigma-Aldrich, St. Louis, MO; diluted 1:10,000 in dilution buffer) was added to each well and incubated for 30 min at 37 °C.

For color development, 90 µL of TMB substrate (cat. #T8665, Sigma-Aldrich, St. Louis, MO) was added to each well and incubated at room temperature in the dark for 5–15 min. The reaction was stopped by adding 45 µL of stop solution (2 M H_2_SO_4_, Honeywell, Perth, Australia, cat. #00646), and absorbance was measured at 450 nm using a Multiskan FC Microplate Spectrophotometer (Thermo Fisher Scientific, USA).

### 2.9. Poliovirus RNA Extraction

Poliovirus RNA was extracted from PV samples using QIAamp Viral RNA Mini Kit (cat #52904, Qiagen, Germantown, MD, USA) according to the manufacturer’s instructions. The concentration of extracted viral RNA samples was determined by using a spectrophotometer at 260 nm (iMark microplate absorbance reader, BioRad, Singapore).

### 2.10. Numerical and Statistical Analysis of Data

All calculations were performed by using standard formulas in Microsoft Excel (linear regression, standard deviation, etc.). To demonstrate antibody specifity for the D- and H- antigens, graphs were plotted showing the mean absorbance at 450 nm (minus background) versus reverse dilution of antigens. All data are presented as averages with standard deviation. Multiple comparisons in two-way ANOVA were used to compare the differences between viruses in each specific dilution in ELISA with the scientific software GraphPad Prism version 8.2.1.

### 2.11. Biosafety and Biosecurity Measures

FSASI “Chumakov FSC R&D IBP RAS” (Institute of Poliomyelitis), Moscow, Russia, is an OPV producer and scientific research institute. It is fully accredited by the national authorities for work with BSL 1–3 agents. Additionally, it has a Certificate of participation in poliovirus containment (RUS-CP-20191202-007) issued by GCC.

## 3. Results

### 3.1. Isolation and Characterization of D- and H-Antigen Fractions

**Figure 1:**
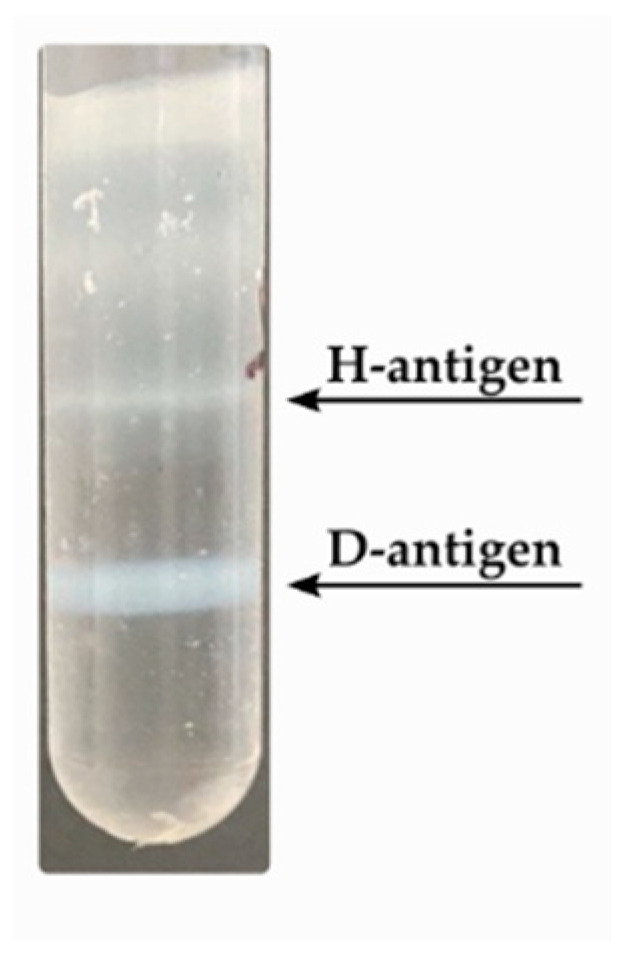
Visible bands in the gradient after ultracentrifugation in a cesium chloride density gradient: the lower band corresponding to D-antigen and the upper band to H-antigen.

To generate specific antibodies, D- and H- antigens were isolated from a Vero cell-derived virus suspension using cesium chloride (CsCl) gradient ultracentrifugation (see Materials and Methods Section 2.1). Following ultracentrifugation, two distinct bands were observed: the upper band corresponding to the H-antigen and the lower band to the D-antigen (Figure 1). The quality and identity of these fractions were assessed using multiple methods, including transmission electron microscopy (TEM), SDS-PAGE analysis of capsid protein composition, and spectrophotometric detection of viral genomic RNA.

Electron microscopy revealed that the upper band predominantly contained empty viral capsids, which lack genomic RNA and correspond to the H-antigen. These structures appear as spherical particles with a central electron-lucent area. In contrast, the lower band was enriched in mature, genome-containing virions, representing the D-antigen.

**Figure 2:**
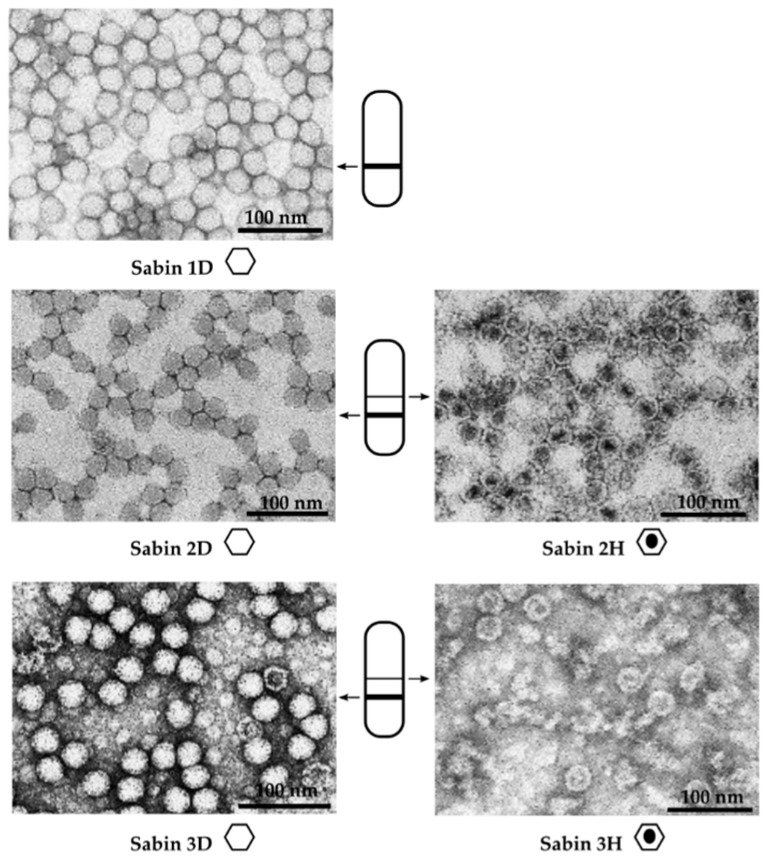
Transmission electron microscopy of purified antigen fractions from Sabin poliovirus strains types 1, 2, and 3. Panels show D-antigen (mature virions) and H-antigen (empty capsids) fractions for each strain. Symbols: white hexagon—mature (genome-containing) viral particles; hexagon with central black oval—empty (genome-free) viral particles. Thick line—the lower band corresponding to D-antigen; thin line—the upper band corresponding to H-antigen. Scale bars: 100 nm.

Both D- and H-antigens were successfully isolated from Sabin type 2 and type 3 strains, whereas only the D-antigen could be obtained from the Sabin type 1 strain (Figure 2).

**Figure 3:**
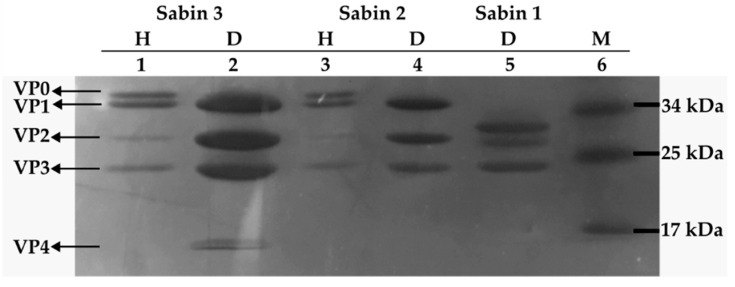
Virions separated in SDS-PAGE followed by Coomassie blue staining. Lane 1 and 2, Sabin 3H and Sabin 3D, respectively; Lane 3 and 4, Sabin 2H and Sabin 2D, respectively; Lane 5, Sabin 1; Lane 6, AceColor Prestained Protein Ladder (ACE Biolabs, Taoyuan, Taiwan, cat #A1054).

The fractions obtained after ultracentrifugation were further analyzed by SDS-PAGE followed by Coomassie Brilliant Blue staining. As shown in Figure 3, the D-antigen fraction was resolved into the four structural capsid proteins: VP1, VP2, VP3, and VP4, consistent with the composition of mature virions. Notably, VP4 was detectable only at relatively high antigen concentrations, reflecting its lower abundance in the capsid.

In contrast, the H-antigen fraction yielded VP0 (the precursor of VP2 and VP4), along with VP1 and VP3, indicating the presence of immature, genome-free viral particles. A faint VP2 band observed in the H-antigen preparation suggested a minor contamination with D-antigen.

To assess the presence of genomic RNA in the viral particles, total RNA was extracted from the purified D- and H-antigen fractions and quantified spectrophotometrically (see Materials and Methods Section 2.9). As presented in Table 1, viral RNA was detected exclusively in the D-antigen samples, whereas the H-antigen fractions contained no measurable RNA (Sabin type 2 and 3).

From the Sabin strain type 1, only D-antigen was obtained, so the H antigen was obtained by heat treatment (56 °C for 1 h). However, during heat treatment, it is assumed that RNA comes out of the capsid. Thus, RNA will also be detected in the H-antigen sample obtained by heat treatment (Table 1, Sabin 1H (heat treatment)). To clearly demonstrate that Sabin strain type 1 H-antigen (obtained by heat treatment) represents capsids without RNA, we treated some of the samples with RNase to remove the RNA that came out of the capsid into solution. As shown in Table 1, after treating with RNase, no measurable RNA is observed (Table 1, Sabin 1H (heat treatment, RNAse treatment)). As a control, the D-antigen was also treated with RNase and RNA was detected, which confirms the integrity of the D-antigen capsid. These results are consistent with the identification of D-antigen as mature, genome-containing virions, and H-antigen as immature, empty capsids.

### 3.2. Evaluation of Serotype Specificity of ELISA Tests for Quantification of Poliovirus D-Antigens

The purified D- and H- antigens were used to immunize rabbits and the polyclonal antibodies were obtained. The neutralizing antibody titers obtained against the Sabin strains: 1:2048—Sabin type 1 poliovirus; more than 1:2048—Sabin type 2 poliovirus; 1:2048—Sabin type 3 poliovirus.

Five ELISA test systems were developed using a sandwich-format design. In each assay, polyclonal rabbit antibodies raised against poliovirus D- and H-antigens were immobilized on ELISA plate wells and served as capture antibodies. Detection was performed using the same polyclonal antibodies, conjugated to (+)-biotin N-hydroxysuccinimide ester (Table 2).

**Figure 4:**
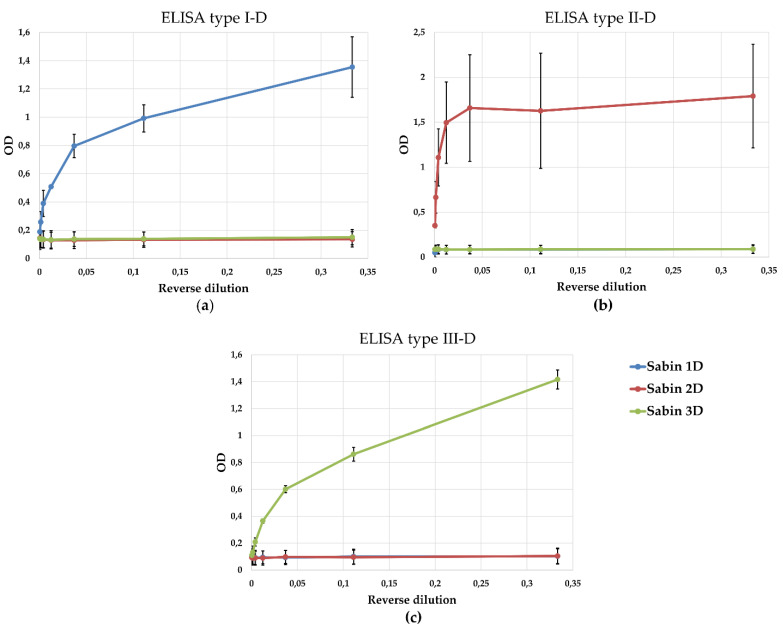
Specificity of ELISA tests for the detection of D-antigens of poliovirus Sabin strains types 1, 2, and 3. Quantitative detection of D-antigens using (**a**) ELISA type I-D; (**b**) ELISA type II-D; and (**c**) ELISA type III-D. Curves: blue—D-antigen of Sabin type 1; red—D-antigen of Sabin type 2; green—D-antigen of Sabin type 3. Data are presented as mean ± standard deviation; *n* = 3 independent experiments.

To evaluate the serotype specificity of the developed ELISA systems, we compared optical density (OD) values obtained for D-antigens of poliovirus Sabin strains types 1, 2, and 3, purified by cesium chloride gradient centrifugation. As shown in Figure 4, each assay exhibited a significantly higher OD for the homologous D-antigen compared to heterologous serotypes. These findings confirm the high serotype specificity of the ELISA systems for the quantitative detection of poliovirus D-antigens. We also confirmed that antibodies to Sabin do not recognize other enteroviruses (FSASI “Chumakov FSC R&D IBP RAS” (Institute of Poliomyelitis)) (Supplemental Figure S2).

### 3.3. Checking the Specificity of the ELISA Tests to Determine D- and H-Antigens of Poliovirus Sabin Strain Type 1,2,3

**Figure 5:**
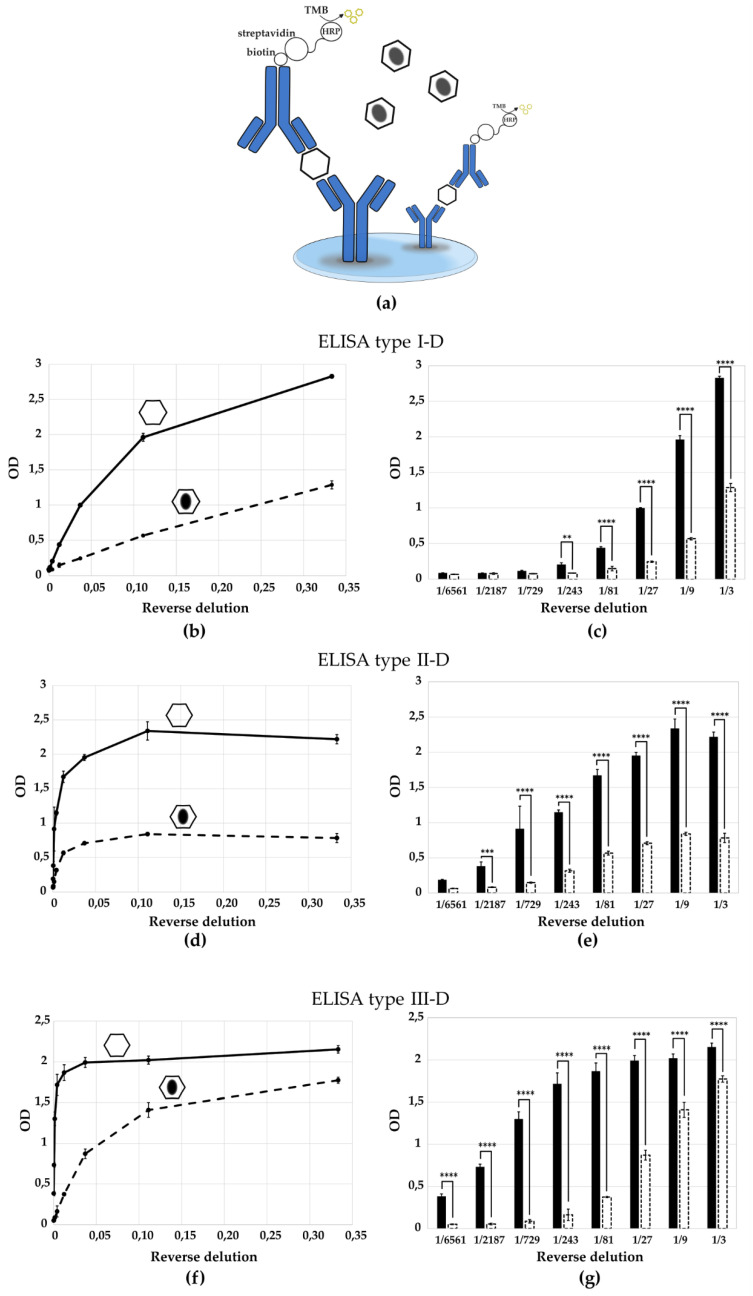
Specificity of the ELISA test to determine D- and H-antigens. Schematic representation of operating principle of the ELISA test systems for recognition of (**a**) D-antigen. Quantitative determination of D-antigen (purified in a cesium gradient) and H-antigen (purified in a cesium gradient for Sabin type 2, 3; produced by thermal treatment for Sabin type 1) of poliovirus strain Sabin (**b**,**c**) type 1 by ELISA type I-D; (**d**,**e**) type 2 by ELISA type II-D; (**f**,**g**) type 3 by ELISA type III-D. Hexagon, solid line and black bar—D-antigen; hexagon with a black oval in the center, dashed line and dashed bar—H-antigen. Results are presented as mean with standard deviation. Number of independent experiments, *n* = 3. **, *p* < 0.01; ***, *p* < 0.001, ****, *p* < 0.0001 according to the Multiple Comparisons for Two-Way ANOVA. Columns in the chart without a *p*-value indicate that the observed differences between the variables are not statistically significant.

**Figure 6:**
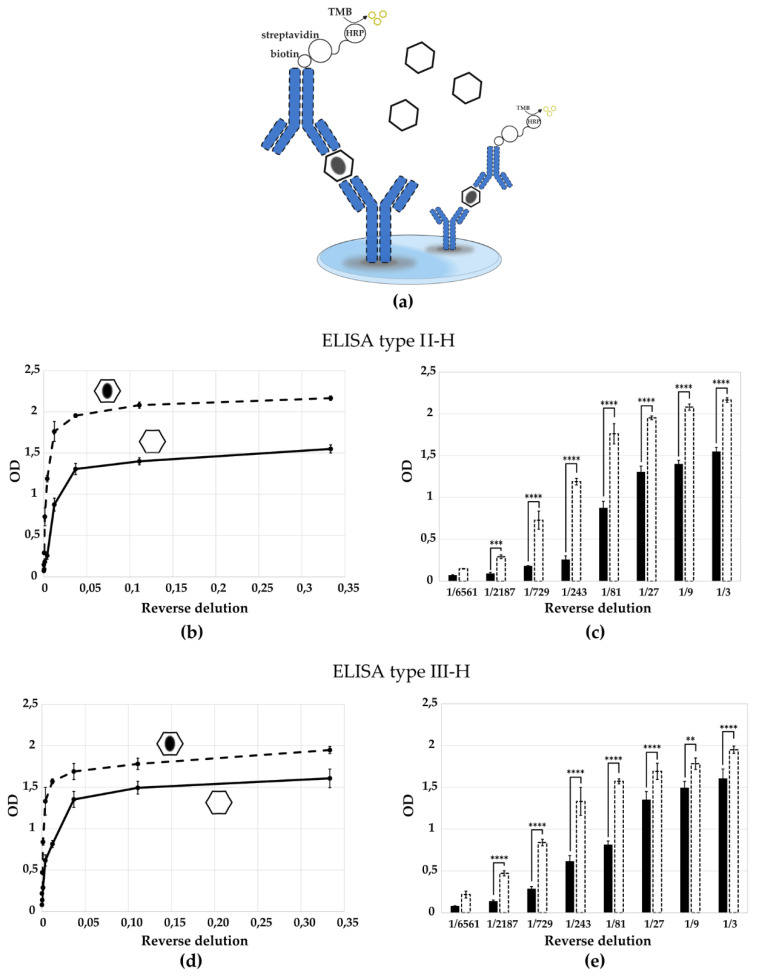
Specificity of the ELISA test to determine D- and H-antigens. Schematic representation of operating principle of the ELISA test systems for recognition of (**a**) H-antigen. Quantitative determination of D-antigen (purified in a cesium gradient) and H-antigen (purified in a cesium gradient for Sabin type 2, 3; produced by thermal treatment for Sabin type 1) of poliovirus strain Sabin (**b**,**c**) type 2 by ELISA type II-H; (**d**,**e**) type 3 by ELISA type III-H. Hexagon, solid line and black bar—D-antigen; hexagon with a black oval in the center, dashed line and dashed bar—H-antigen. Results are presented as mean with standard deviation. Number of independent experiments, *n* = 3. **, *p* < 0.01; ***, *p* < 0.001, ****, *p* < 0.0001 according to the Multiple Comparisons for Two-Way ANOVA. Columns in the chart without a *p*-value indicate that the observed differences between the variables are not statistically significant.

To assess the specificity of the generated antibodies, we compared the optical density (OD) values obtained for purified D- and H-antigens using the ELISA test system. H-antigens were purified via cesium chloride gradient centrifugation from Sabin type 2 and 3; for Sabin type 1 H-antigens were produced by thermal treatment (56 °C for 1 h) because only D-antigens were obtained from the Sabin type 1 strain). The results are presented in Figure 5 and Figure 6. Antibodies raised against the D-antigen exhibited significantly higher reactivity toward the D-antigen compared to the H-antigen, confirming their specificity (Figure 5b,d,f). Conversely, antibodies generated against the H-antigen preferentially recognized the H-antigen, showing reduced binding to the native D-antigen (Figure 6b,d). According to the multiple comparisons for two-way ANOVA the difference in the optical density value between the antigen types is statistically significant for most virus dilutions, which indicates that one curve is significantly different from the other (Figure 5c,e,g and Figure 6c,e). This confirms the specificity of the obtained ELISA tests.

Furthermore, D- and H-antigen specificity of ELISA test systems was assessed by testing experimental vaccine sample of trivalent sIPV (FSASI “Chumakov FSC R&D IBP RAS” (Institute of Poliomyelitis)) in native form and H-form. Each ELISA test detected only antigen-specific binding (Supplemental Figure S3). Sample of trivalent sIPV had been converted to H-antigen by heating at 56 °C for 1 h.

### 3.4. Evaluation of ELISA Specificity for Distinguishing D- and H-Antigens in the sIPV 17/160 Standard

**Figure 7:**
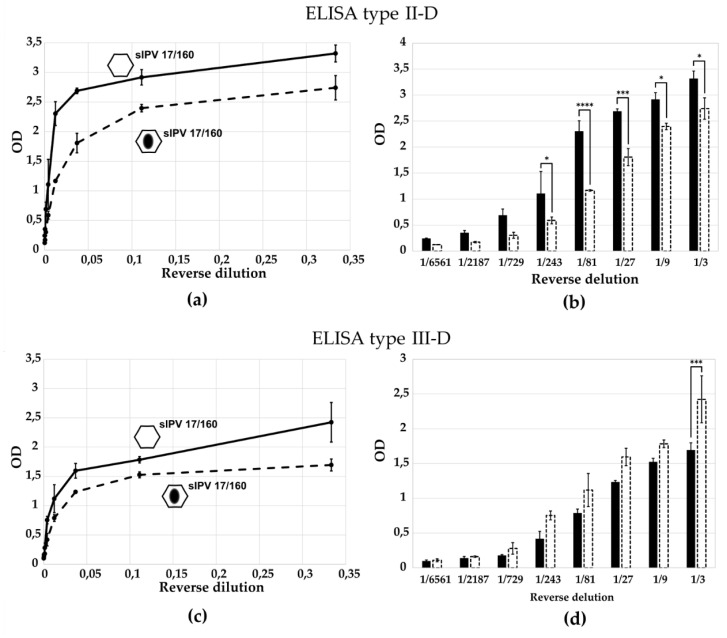
Specificity of ELISA test for the detection of D- and H-antigens in the 1st sIPV 17/160 standard. Quantitative assessment of D-antigens (1st sIPV 17/160) and H-antigens (1st sIPV 17/160, heat-treated for 1 h at 56 °C) was performed using: (**a**,**b**) ELISA type II-D, (**c**,**d**) ELISA type III-D. D-antigens are represented by hexagons, solid line and black bar; H-antigens by hexagons with a black oval, dashed line and dashed bar. Data are presented as mean with standard deviation from three independent experiments (*n* = 3). *, *p* < 0.05; ***, *p* < 0.001, ****, *p* < 0.0001 according to the Multiple Comparisons for Two-Way ANOVA. Columns in the chart without a *p*-value indicate that the observed differences between the variables are not statistically significant.

**Figure 8:**
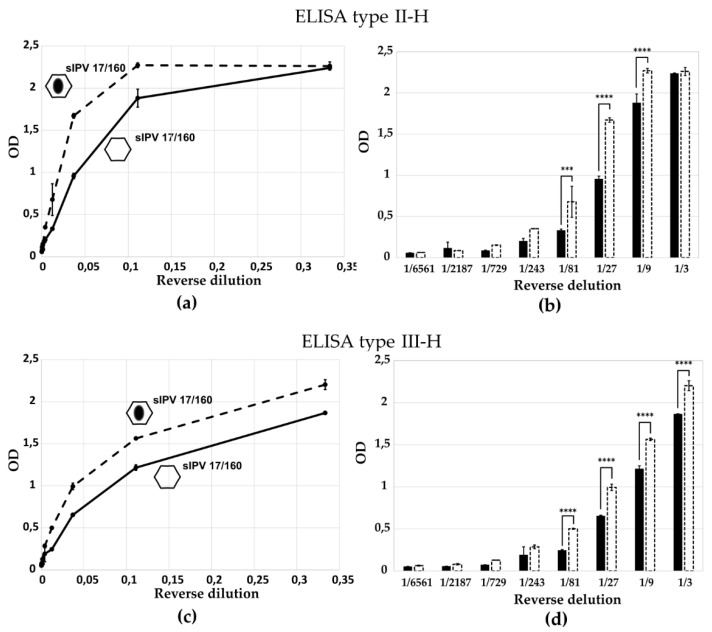
Specificity of ELISA test for the detection of D- and H-antigens in the 1st sIPV 17/160 standard. Quantitative assessment of D-antigens (1st sIPV 17/160) and H-antigens (1st sIPV 17/160, heat-treated for 1 h at 56 °C) was performed using: (**a**,**b**) ELISA type II-H, (**c**,**d**) ELISA type III-H. D-antigens are represented by hexagons, solid line and black bar; H-antigens by hexagons with a black oval, dashed line and dashed bar. Data are presented as mean with standard deviation from three independent experiments (*n* = 3). ***, *p* < 0.001, ****, *p* < 0.0001 according to the Multiple Comparisons for Two-Way ANOVA. Columns in the chart without a *p*-value indicate that the observed differences between the variables are not statistically significant.

To evaluate antibody specificity, we compared the optical density (OD) values obtained in ELISA tests using the International Standard for Sabin Inactivated Poliovirus Vaccine (1st sIPV 17/160, NIBSC, Potters Bar, UK) and the same standard subjected to heat treatment at 56 °C for 1 h, which induces antigenic transition: formation of H-antigens. The results are presented in Figure 7 and Figure 8. As shown in Figure 7a,b, antibodies raised against the D-antigen of Sabin type 2 exhibited higher reactivity toward the native (D-antigen-containing) standard compared to the heat-treated H-antigen preparation, confirming their specificity. Conversely, antibodies generated against the H-antigen preferentially recognized the heat-treated (H-antigen) form over the native standard, as illustrated in Figure 8a–d. According to two-way ANOVA with multiple comparisons, the difference in OD values between D- and H-antigen preparations was statistically significant for the first 3–5 virus dilutions (Figure 7b and Figure 8b,d), indicating that the two curves differ significantly in this range and confirming the specificity of the developed ELISA tests. For ELISA type III-D, however, a statistically significant difference was observed only at the highest antigen concentration (Figure 7c,d), suggesting lower discriminatory capacity in this format.

### 3.5. Differential Recognition of IPV Standards by Sabin-D-Specific Antibodies

**Figure 9:**
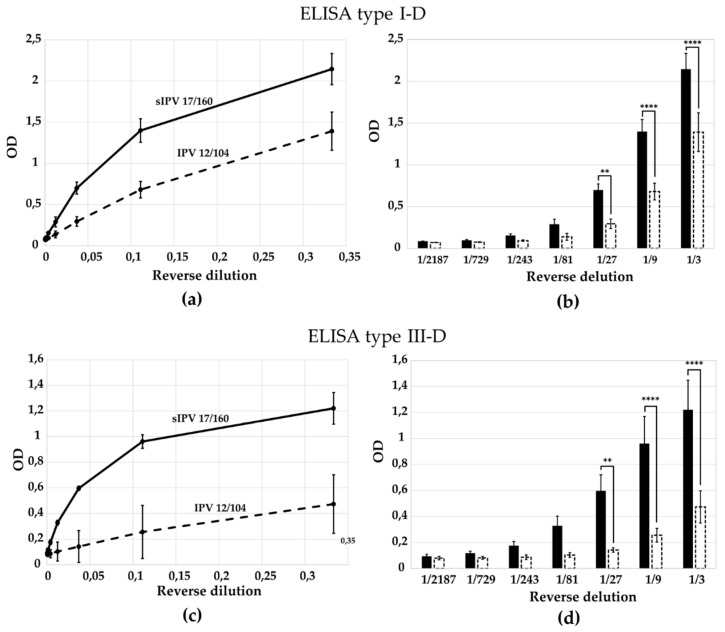
Quantitative determination of D-antigens in the 1st International Standard for Sabin IPV (sIPV 17/160) and the 2nd International Standard for conventional wild-type IPV (cIPV 12/104) using (**a**,**b**) ELISA type I-D and (**c**,**d**) ELISA type III-D. Solid line and black bar represent sIPV 17/160; dashed line and dashed bar represent IPV 12/104. Data are shown as mean ± standard deviation from three independent experiments (*n* = 3). Statistical significance was evaluated using two-way ANOVA with multiple comparisons (**, *p* < 0.01; ****, *p* < 0.0001). Bars without *p*-value labels indicate no statistically significant differences between compared groups.

We evaluated the performance of our D-antigen-specific ELISA tests using two international standards: the 2nd International Standard for wild-type IPV (IPV 12/104, NIBSC, UK) and the 1st International Standard for Sabin Inactivated Poliovirus Vaccine (sIPV 17/160, NIBSC, UK). The results, shown in Figure 9, demonstrate that ELISAs based on antibodies specific to Sabin poliovirus strains detect the sIPV 17/160 standard more efficiently than the wild-type-based 12/104 standard (Figure 9a,c). Despite comparable levels of D-antigen content for types 1 and 3 in both standards, the ELISA signals were significantly different. According to two-way ANOVA with multiple comparisons, statistically significant differences were observed in OD values for the first 3–5 virus dilutions (Figure 9b,d), confirming preferential recognition of the homologous Sabin antigen. These findings indicate antigenic differences between sIPV and wild-type IPV preparations and highlight the importance of using matched antigen–antibody pairs for accurate quantification.

The results, shown in Figure 9, demonstrate that ELISAs based on antibodies specific to Sabin poliovirus strains detect the sIPV 17/160 standard more efficiently than the wild-type-based 12/104 standard (Figure 9a,c). Despite comparable levels of D-antigen content for types 1 and 3 in both standards (17/160 was established in 2018 with an assigned potency using the WHO IS for cIPV 12/104 as reference of 239, 136 and 237 of D-Antigeng Units/mL for poliovirus type 1, 2 and 3 respectively), the ELISA signals were significantly different. According to two-way ANOVA with multiple comparisons, statistically significant differences were observed in OD values for the first 3–5 virus dilutions (Figure 9b,d), confirming preferential recognition of the homologous Sabin antigen. These findings indicate antigenic differences between sIPV and wild-type IPV preparations and highlight the importance of using matched antigen–antibody pairs for accurate quantification.

### 3.6. Calibration Curves and D-Antigen Content in Experimental Vaccines Samples Based on Sabin Strains by Developed ELISA Tests

**Figure 10:**
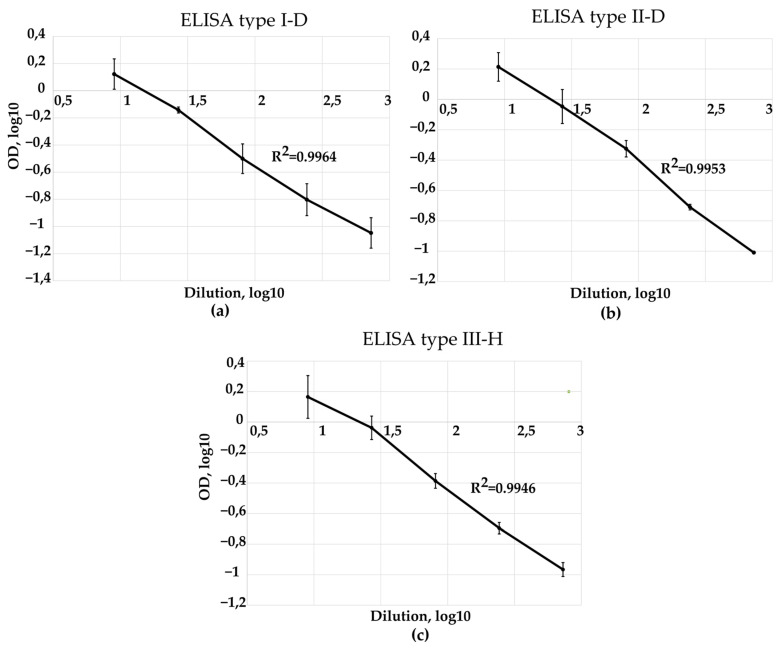
Standard curves of sIPV 17/160 (log-log transformed raw data) using (**a**) ELISA type I-D, (**b**) ELISA type II-D and (**c**) ELISA type III-D. Data are shown as mean ± standard deviation from three independent experiments (*n* = 3).

**Figure 11:**
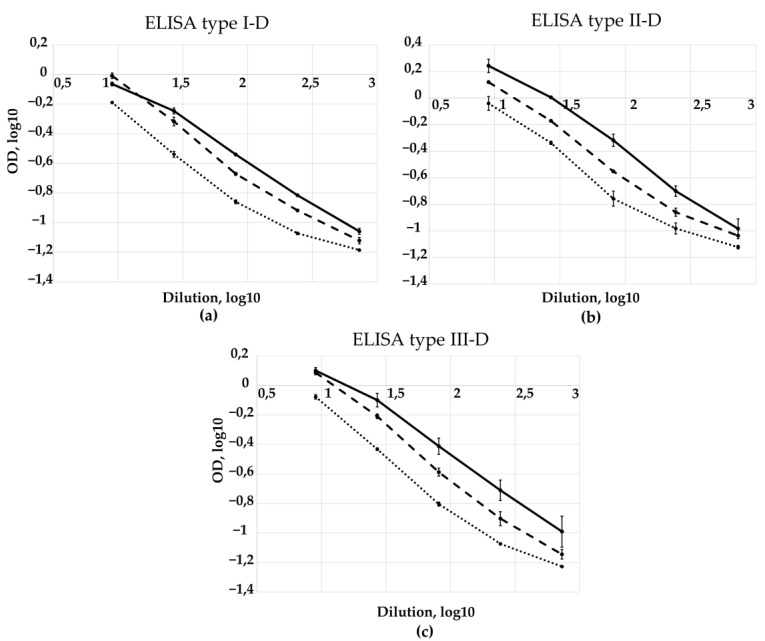
Standard curves of sIPV 17/160 and experimental vaccine samples of trivalent sIPV (log-log transformed raw data) using (**a**) ELISA type I-D, (**b**) ELISA type II-D and (**c**) ELISA type III-D. Solid lines represent sIPV 17/160; dashed lines represent sample 1; dotted lines represent sample 2. Data are shown as mean ± standard deviation from three independent experiments (*n* = 2).

Calibration curves were constructed for each of the developed ELISA tests (type I-D, II-D, III-D) using sIPV 17/160 standard and experimental vaccine samples of trivalent sIPV (FSASI “Chumakov FSC R&D IBP RAS” (Institute of Poliomyelitis)). These curves were generated by analyzing serial three-fold dilutions of the standard, starting with a 1:9 dilution. Representative calibration curves for each serotype are shown in Figure 10 and Figure 11. Raw optical density values were plotted against the corresponding dilutions on a log-log scale. Serial dilutions of the standard were selected in the optimal range that provided the highest reading no more than 2.0 OD_450_ and the lowest dilution that gave a signal more than 2 times different from the blank.

The determination coefficient exceeds 0.99 (Figure 10); therefore, there is a direct linear relationship between the measured optical density and the concentration of D-antigen in the standard.

The performance of the developed ELISA systems for quantification of D-antigen was assessed using experimental samples of trivalent sIPV with sIPV 17/160 as the reference standard. Representative calibration curves obtained for each serotype (Figure 11a–c) demonstrate a linear and parallel response across the tested dilution range, indicating the suita-bility of the assays for quantitative analysis. The measured D-antigen contents in experimental samples of trivalent sIPV are presented in Table 3.

For experimental vaccines samples of trivalent sIPV the coefficients of variation (CV) were not exceeding 8.3% for any of the serotypes tested. These results confirm that the ELISA assays provide precise and reproducible determination of D-antigen content, with inter- and intra-assay variability remaining well below the generally accepted acceptance cri-terion of 15%. Thus, the established ELISA systems can be applied for reliable quantification of antigen content in sIPV preparations and hold promise for implementation in vaccine quality control.

## 4. Discussion

The efficacy of poliovirus vaccines critically depends on the precise quantification of the immunogenic D-antigen, which is responsible for eliciting neutralizing antibodies. Accurate measurement of the D-antigen is essential for vaccine evaluation, particularly due to its potential conversion into the non-protective H-antigen.

For vaccine quality control, traditionally D-antigen ELISA is used. It is worth mentioning that polyclonal antibody-based ELISA, such as the one developed in our study, and monoclonal antibody-based ELISA (widespread D-antigen detection method) represent two distinct technical paradigms for antigen detection. Each approach carries advantages and limitations that must be carefully considered in the context of vaccine quality control.

Polyclonal antibodies constitute a heterogeneous mixture of immunoglobulin molecules that recognize multiple epitopes across the target antigen, while monoclonal antibodies represent a homogeneous population of immunoglobulins that bind to a single, specific epitope. This broad recognition profile of polyclonal antibodies confers a significant advantage in detecting potentially diverse antigenic configurations that may arise from slight variations in vaccine preparation or viral strain differences. Diversity in polyclonal antibodies makes the detection system more robust against minor structural variations in the D-antigen, which is particularly valuable when working with Sabin strains that may exhibit subtle structural differences compared to wild-type poliovirus strains. In contrast, monoclonal antibody-based ELISA offer specificity for a single epitope, providing precision in targeting a defined antigenic determinant. This high specificity can be advantageous when targeting a functionally critical epitope.

Our polyclonal antibody-based ELISA offers substantial cost advantages in initial development. In contrast, the initial development phase for monoclonal antibodies usually takes several months and requires significant personnel resources for hybridoma generation, screening, and cloning. However, once developed, monoclonal antibodies can be produced indefinitely from stable cell lines.

One of the most significant challenges associated with polyclonal antibody-based ELISA is the inherent batch-to-batch variability. Each animal immunization event produces a unique antibody repertoire, even when using identical antigen preparations and immunization protocols. This variability requires strict quality control measures and batch validation protocols to minimize this variability.

Each approach offers distinct advantages that must be weighed against specific application requirements, resource constraints, and performance priorities. Our polyclonal antibody-based ELISA demonstrates particular strengths in detection robustness and cost-effectiveness for initial implementation, making it especially suitable for quality control applications where broad antigen recognition and rapid method development are prioritized.

Despite the fact that the primary method for detecting and quantifying D-antigen in poliovirus vaccine production is the ELISA alternative methods are being developed. For example, surface plasmon resonance (SPR) technology measures binding interactions in real time without the need to label the interacting molecules [28].

Among available methods, ELISA remains the gold standard for D-antigen quantification owing to its high sensitivity and specificity, ensuring the reliability of vaccine potency assessment.

In this study, we developed and characterized a panel of ELISA systems for the detection and differentiation of poliovirus D- and H-antigens derived from Sabin strains. Our approach was based on the purification of structurally distinct viral particles from Vero cell-derived preparations using cesium chloride gradient ultracentrifugation. The D-antigen fractions corresponded to mature, genome-containing virions, while the H-antigen fractions contained immature, genome-free empty capsids, as confirmed by electron microscopy, SDS-PAGE profiling of capsid proteins, and RNA quantification.

The ELISA systems based on polyclonal antibodies raised against D- or H-antigens of poliovirus Sabin strains exhibited high serotype and antigen-form specificity. Each D-antigen-specific ELISA (types I-D, II-D, III-D) demonstrated significantly stronger reactivity with its homologous antigen compared to heterologous serotypes, indicating minimal cross-reactivity.

ELISA targeting D-antigens clearly discriminated between native and H-antigen (heat-treated: Supplemental Figure S3 and Figure 7) or between native and H-antigen (empty capsid: Figure 5) preparations, confirming their ability to selectively detect immuno-genic virions.

ELISA test systems constructed with antibodies against H-antigen showed preferential binding to heat-treated (Supplemental Figure S3 and Figure 8) or empty capsid-rich (Figure 6) preparations, while displaying minimal cross-reactivity with D-antigen-containing virions.

It was shown the specificity of the ELISA tests for sIPV 17/160 (from Sabin strains) in form of D-antigen and H-antigen (by the heat treatment). This specificity emphasizes the critical role of D-antigens in eliciting an effective immune reaction, particularly in the context of vaccine development. Furthermore, the heat treatment not only affects the antigenic profile but also raises questions regarding the stability and efficacy of the vaccine. These findings underscore the importance of optimizing vaccine preparation processes to ensure that the immunogenic properties are preserved. It is worth mentioning that for ELISA type III-D test the difference in the optical density value between D- and H-form sIPV 17/160 is statistically significant only for the first virus dilution (Figure 7c,d). Probably the reason is the sufficient stability of Sabin type 3 in sIPV 17/160 and heat treatment at 56 °C for 1 h is not enough for the formation of H-antigens. Therefore, we did not observe a significant difference in this case, likely reflecting the relative thermal stability of Sabin type 3 particles in sIPV 17/160.

Also it was shown greater specificity of the developed ELISA tests in recognizing the standard sIPV 17/160 than in recognizing IPV 12/104. The observed differences in assay highlight the need for tailored potency testing approaches, as traditional assays developed for wild-type IPV may not adequately reflect the potency of sIPV vaccines.

ELISA tests for D-antigen reliably measure the antigen content in samples of trivalent sIPV. The system is satisfactory and can be further used to control vaccine production. However, to accurately quantify D-antigen in the presence of H-antigen (and vice versa) further experiments are needed. Completion of the validation of the obtained ELISA systems for D- and H-antigens is our goal for further research.

Overall, our results demonstrate that the developed ELISA are capable of accurately detecting and distinguishing D- and H-antigen forms of poliovirus across all three Sabin serotypes. They provide a valuable tool for assessing antigen quality and content in Sabin-based IPV formulations and support the need for homologous antigen–antibody systems when evaluating vaccine potency. The ability to selectively detect H-antigens also enables monitoring of non-immunogenic components that may affect vaccine efficacy or stability, thereby contributing to improved quality control and standardization in sIPV production.

## 5. Conclusions

We developed a robust protocol for the purification of poliovirus antigens in both active (D-antigen) and inactive (H-antigen) forms. An optimal immunization schedule was established for rabbits, enabling the production of high-quality polyclonal antibodies suitable for use in ELISA-based quantification of D-antigen in poliovirus vaccines.

A direct “sandwich” ELISA format was selected, and the most effective method for biotinylating IgG was optimized. Subsequent detection using streptavidin–peroxidase provided a highly sensitive assay. Antibody dilutions were optimized to ensure that serum from a single rabbit yields sufficient material for routine ELISA use over several years.

The five ELISA systems developed using these polyclonal rabbit antibodies demonstrated high specificity across all three poliovirus serotypes and reliably distinguished D-antigen from H-antigen forms.

## Figures and Tables

**Table 1:** Concentration of extracted viral RNA in Sabin antigens.

Antigen	Probe Number	Concentration, µg/mL	A260/A280
Sabin 1D	1 2 3	192.8 185.1 214.1	1.99 1.91 1.99
Sabin 1H (heat treatment)	1 2 3	174.9 180.3 177.2	1.96 2.00 1.96
Sabin 1D (RNAse treatment)	1 2 3	136.9 134.3 130.9	1.98 1.89 1.90
Sabin 1H (heat treatment, RNAse treatment)	1 2 3	0.3 * 0.86 * 0.79 *	1.40 0.15 1.15
Sabin 3D	1	590.14	2.09
	2	244.91	2.21
	3	105.70	2.16
Sabin 3H	1	14.09 *	0.74
	2	10.77 *	0.00
	3	7.40 *	1.87
Sabin 2D	1	71	2.15
Sabin 2H	1	1.50 *	1.60

* Below the sensitivity threshold of the spectrophotometer.

**Table 2:** ELISA test systems.

ELISA Type I-D	ELISA Type II-D	ELISA Type III-D	ELISA Type II-H	ELISA Type III-H
D-antigen Sabin type 1	D-antigen Sabin type 2	D-antigen Sabin type 3	H-antigen Sabin type 2	H-antigen Sabin type 3

**Table 3:** D-antigen content in experimental vaccines samples of trivalent sIPV by developed ELISA Systems.

Sample	Average, sDU/mL			%CV		
	ELISA Type I-D	ELISA Type II-D	ELISA Type III-D	ELISA Type I-D	ELISA Type II-D	ELISA Type III-D
Sample 1	31.6	27.5	23.5	0.8	8.3	3.2
Sample 2	63.3	59.3	54.7	6.7	0.2	1.9

## Data Availability

Raw data is available from the authors upon reasonable request.

## Supplementary Materials

The following supporting information can be downloaded at: https://www.mdpi.com/article/10.3390/vaccines13101022/s1, Figure S1: Virus purification scheme. Main stages of purification of D- and H-antigens of poliovirus strain Sabin types 1, 2 and 3, Figure S2: The cross reactivity of the Sabin polioviruses and other main enteroviruses titer-aligned, Figure S3: Specificity of the ELISA test to determine D- and H-antigens (heat treatment) in experimental vaccine samples of trivalent sIPV.
