# Supplementary material for: Generation of Polyclonal Antibodies Against Sabin Poliovirus D- and H-Antigens and Their Application in ELISA
Source: Vaccines (Basel). 2025 Sep 30;13(10):1022. doi: 10.3390/vaccines13101022 (PMC12568119; doi:10.3390/vaccines13101022)
Supplement: Supplementary file 1 [file vaccines-13-01022-s001.zip › Supplementary materials.pdf]

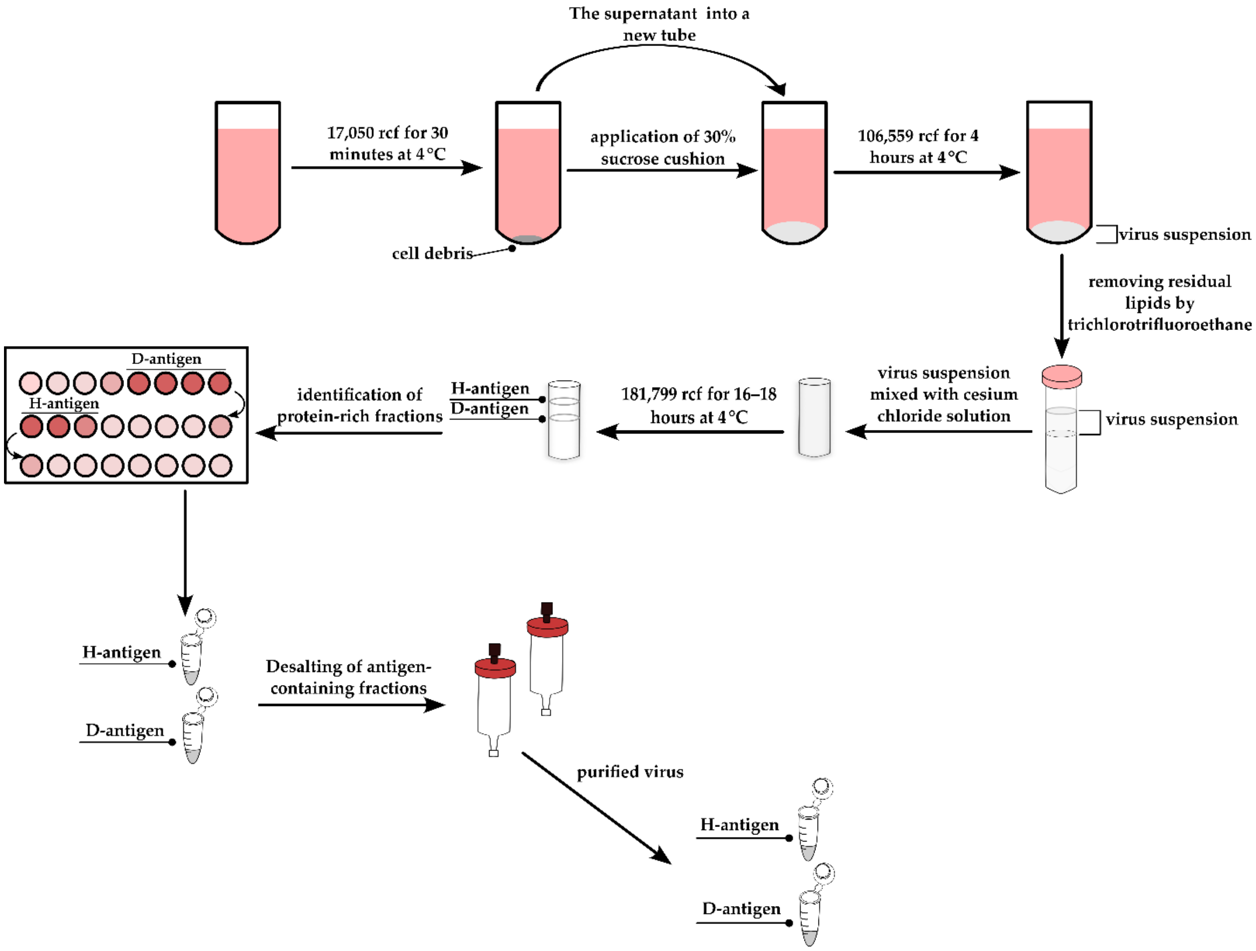

Figure S1. Virus purification scheme. Main stages of purification of D- and H-antigens of poliovirus strain Sabin types 1, 2 and 3.

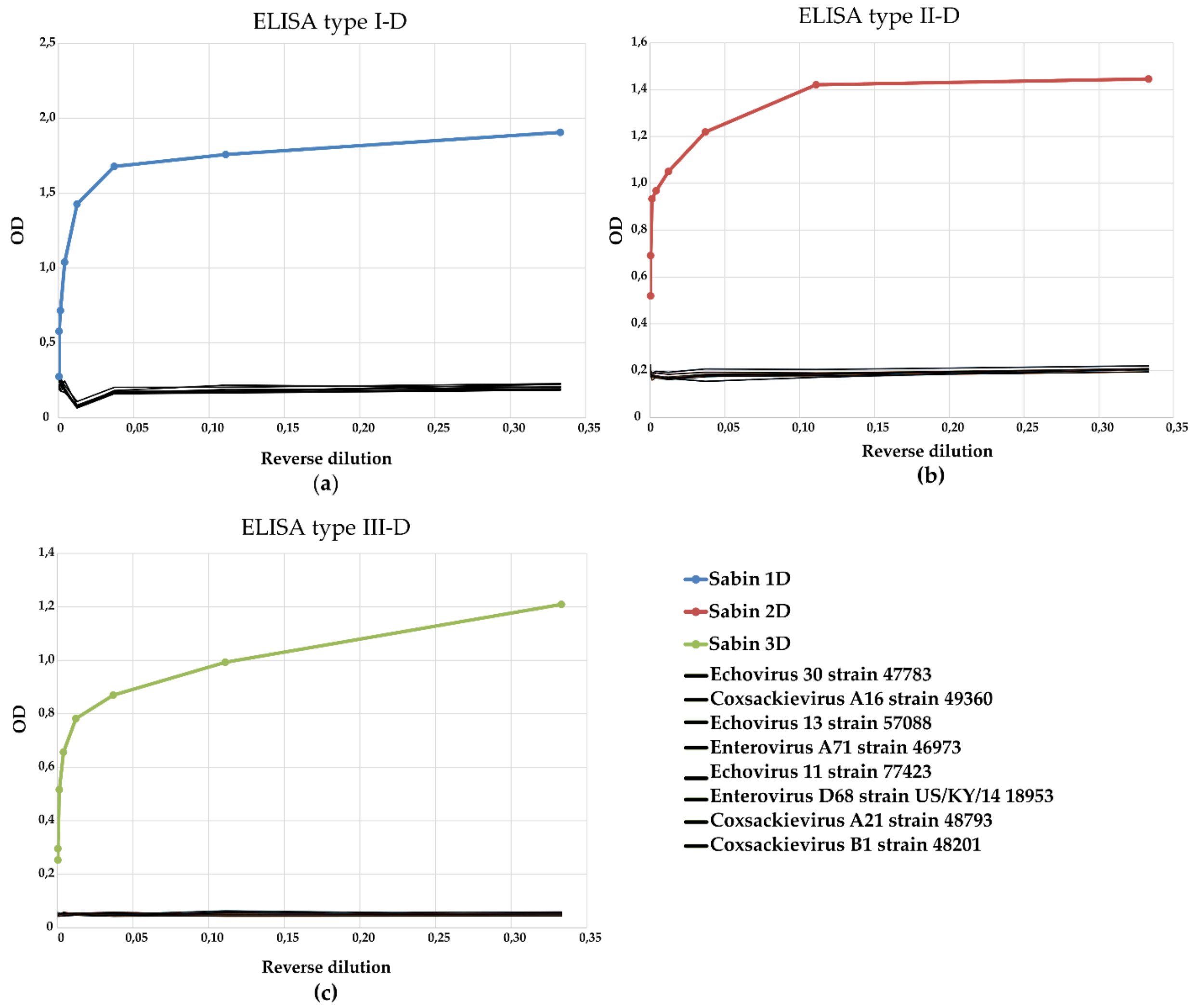

Figure S2. The cross reactivity of the Sabin polioviruses and other main enteroviruses titer-aligned. Quantitative detection of D-antigens using (a) ELISA type I-D; (b) ELISA type II-D; and (c) ELISA type III-D. Curves: blue – D-antigen of Sabin type 1; red – D-antigen of Sabin type 2; green – D-antigen of Sabin type 3, black – main enteroviruses (collection of FSASI "Chumakov FSC R&D IBP RAS" (Institute of Poliomyelitis)).

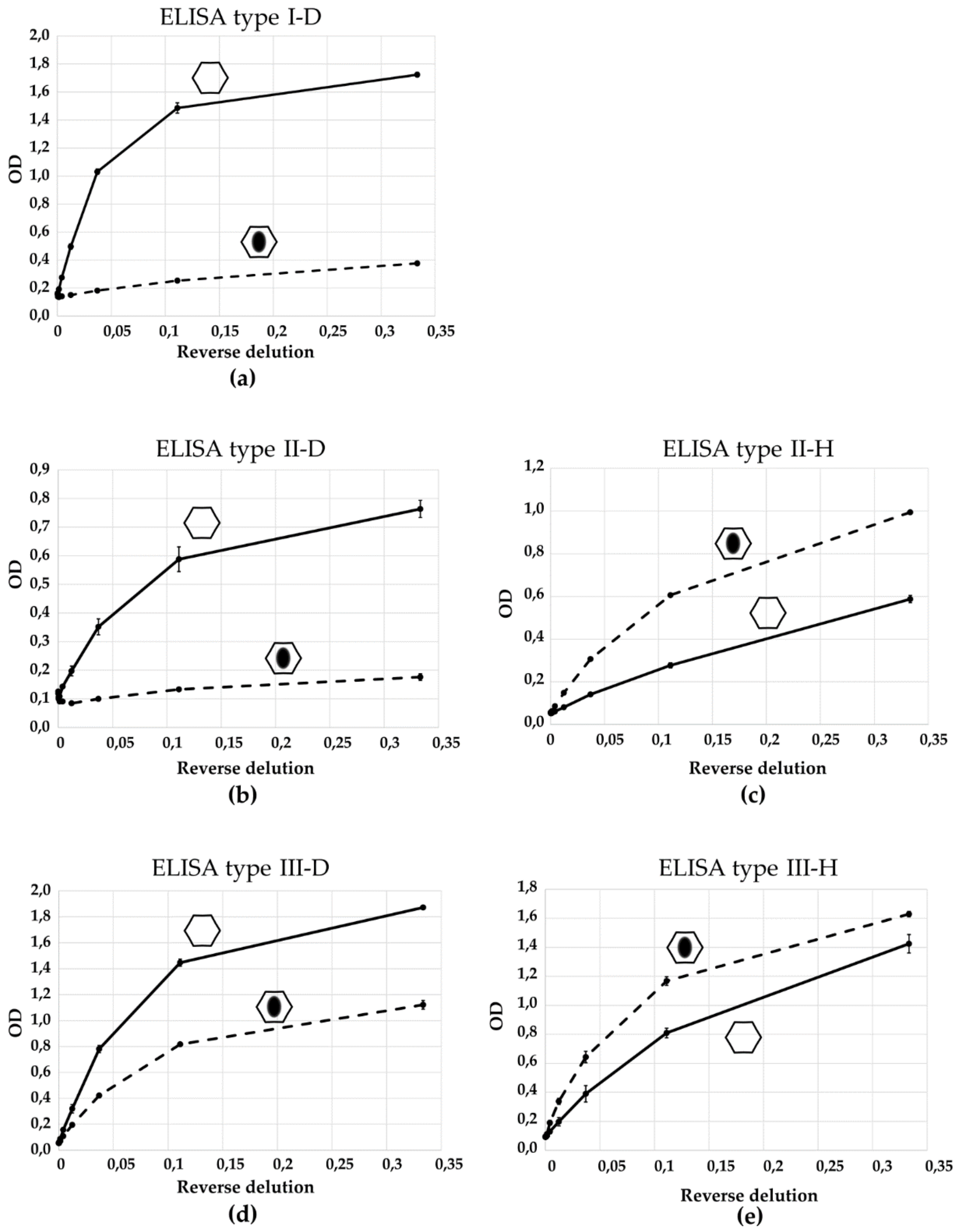

Figure S3. Specificity of the ELISA test to determine D- and H-antigens (heat treatment) in experimental vaccine samples of trivalent sIPV. Quantitative determination of D-antigen (vaccine sample) and H-antigen (heat-treated vaccine sample – 56°C for 1 hour) (a) by ELISA type I-D; (b) by ELISA type II-D; (c) by ELISA type II-H; (d) by ELISA type III-D; (e) by ELISA type III-H. Hexagon, solid line – D-antigen; hexagon with a black oval in the center, dashed line – H-antigen. Results are presented as mean with standard deviation. Number of independent experiments, n = 2.
