# Supplementary figures and images for: Generation of Polyclonal Antibodies Against Sabin Poliovirus D- and H-Antigens and Their Application in ELISA
Source: Vaccines (Basel). 2025 Sep 30;13(10):1022. doi: 10.3390/vaccines13101022 (PMC12568119; doi:10.3390/vaccines13101022)

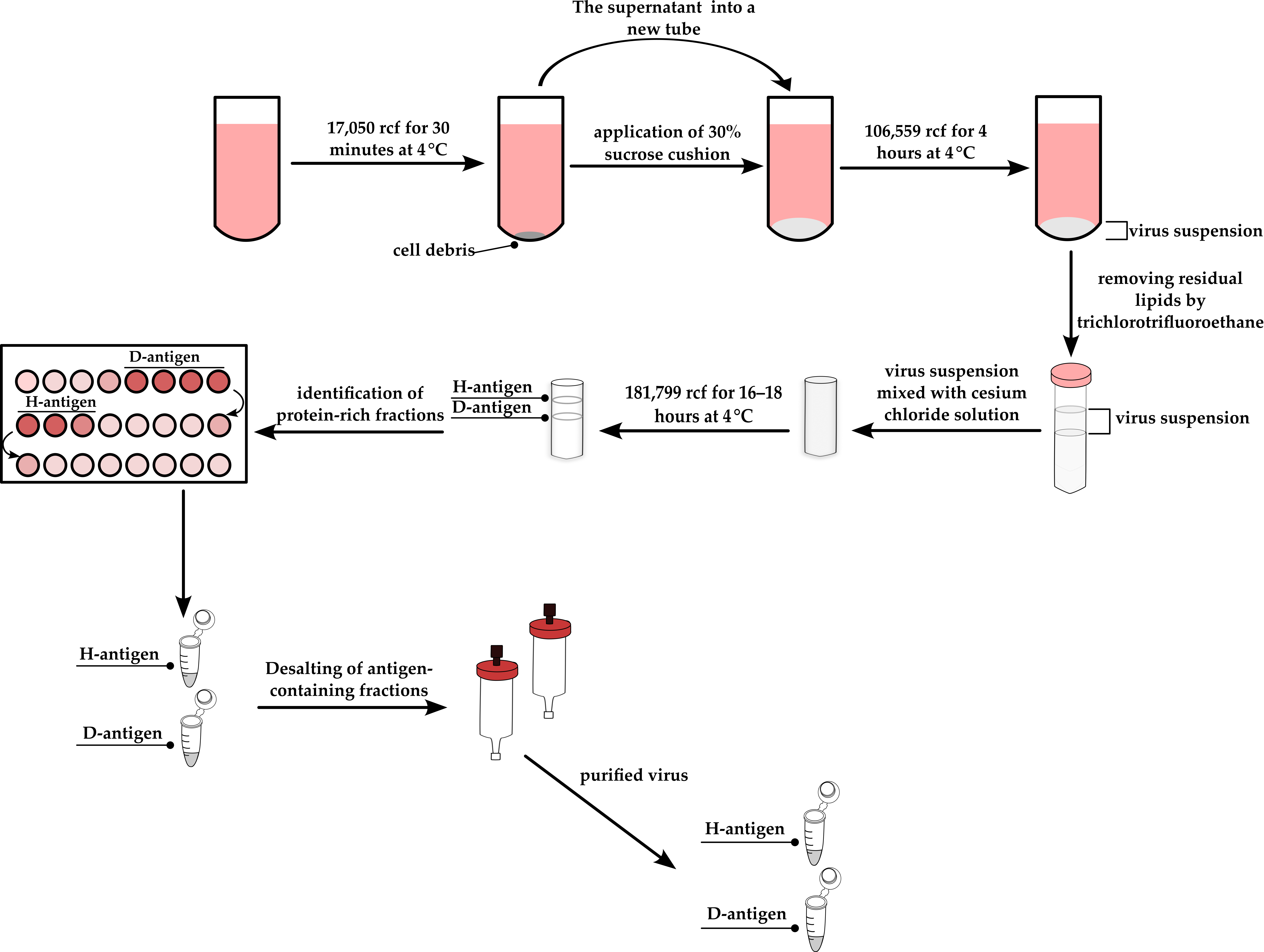

Supplement: Supplementary file 1 [file vaccines-13-01022-s001.zip › Supplemental Figure S1.png]

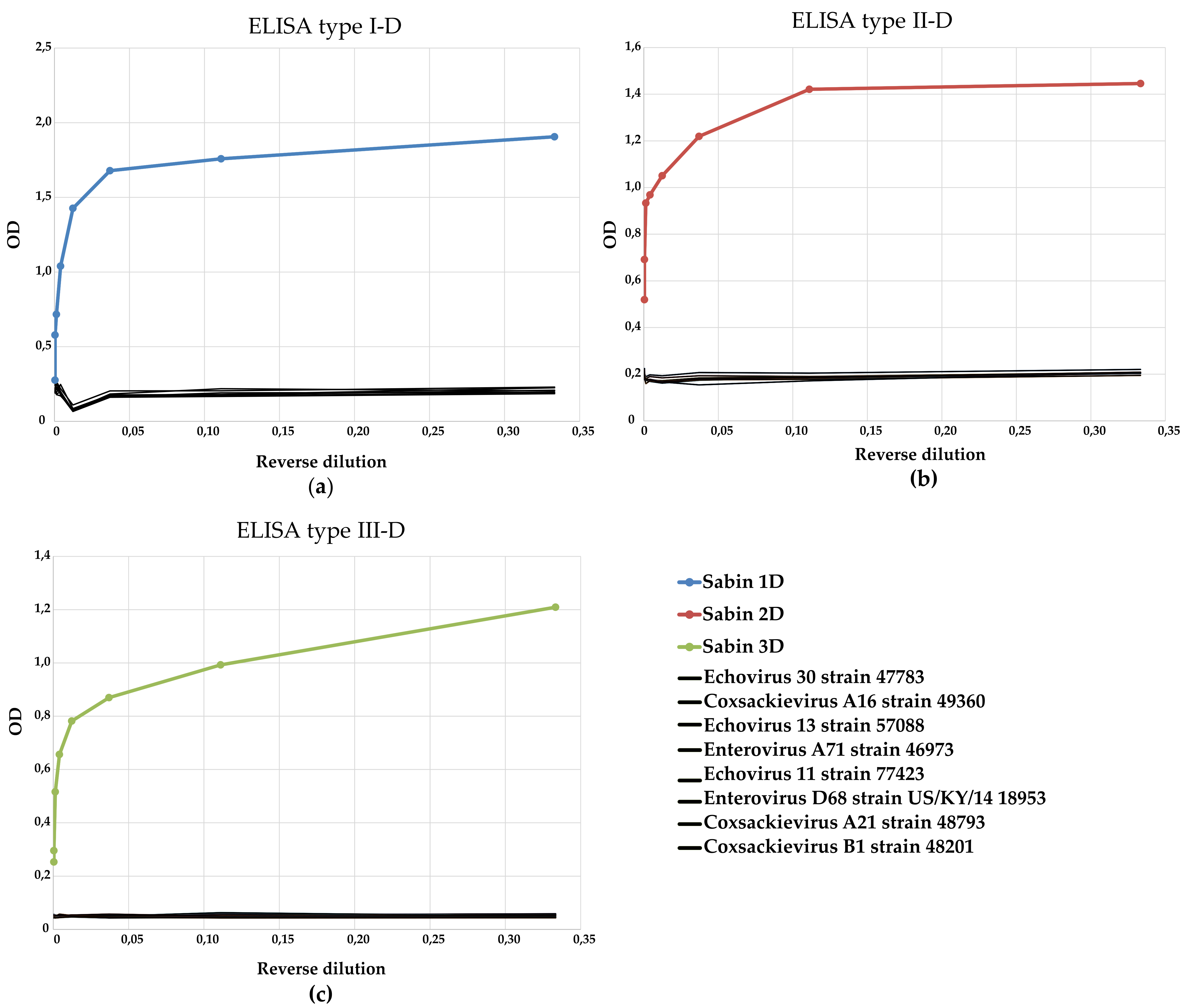

Supplement: Supplementary file 1 [file vaccines-13-01022-s001.zip › Supplemental Figure S2.png]

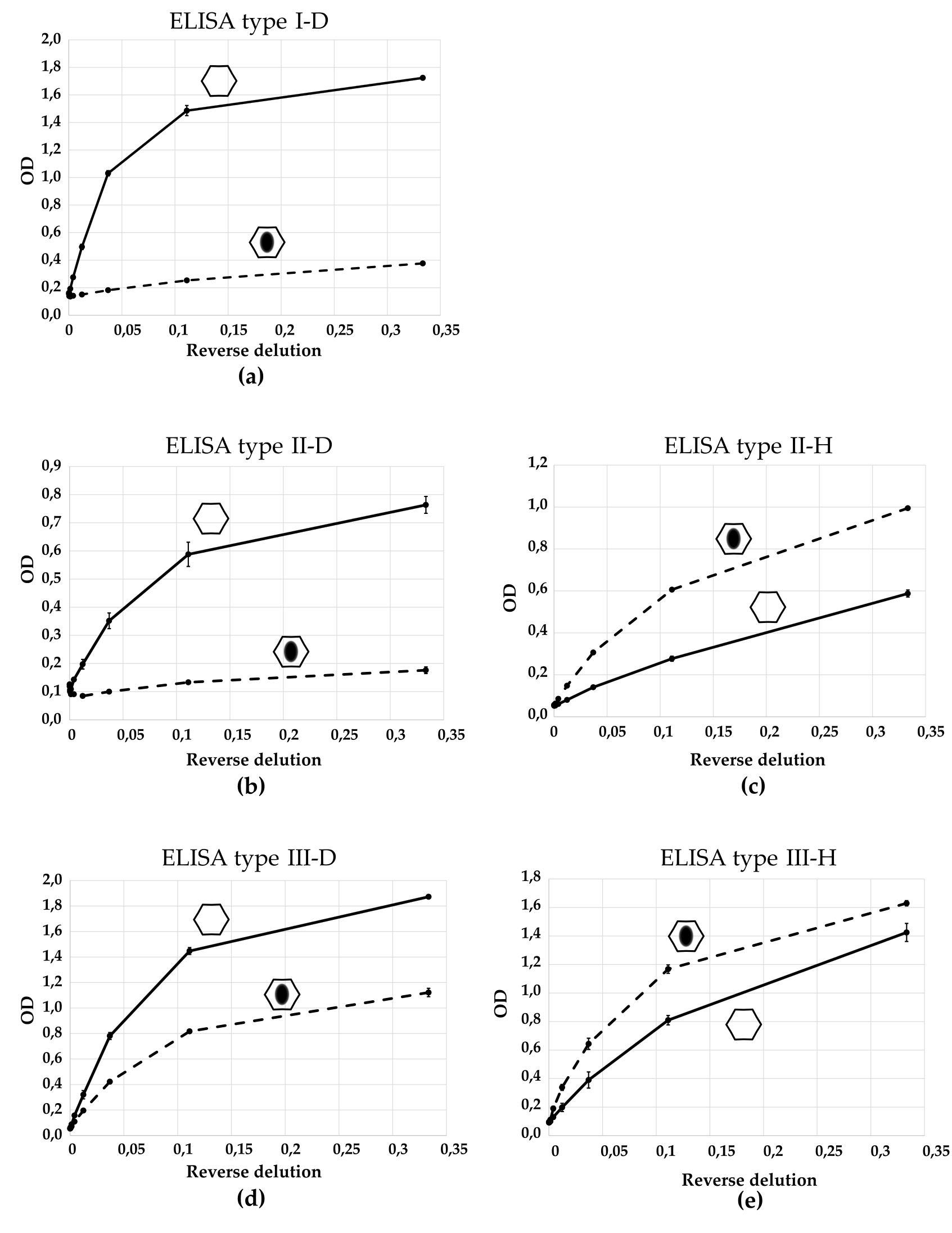

Supplement: Supplementary file 1 [file vaccines-13-01022-s001.zip › Supplemental Figure S3.png]
